# An RNAi screen for conserved kinases that enhance microRNA activity after dauer in *Caenorhabditis elegans*

**DOI:** 10.1093/g3journal/jkae007

**Published:** 2024-01-16

**Authors:** Himal Roka Pun, Xantha Karp

**Affiliations:** Department of Biology, Central Michigan University, Mount Pleasant, MI 48859, USA; Biochemistry, Cell and Molecular Biology Program, Central Michigan University, Mount Pleasant, MI 48859, USA; Department of Biology, Central Michigan University, Mount Pleasant, MI 48859, USA; Biochemistry, Cell and Molecular Biology Program, Central Michigan University, Mount Pleasant, MI 48859, USA

**Keywords:** diapause, dauer, *C. elegans*, microRNA, miRISC, Argonaute, kinase

## Abstract

Gene regulation in changing environments is critical for maintaining homeostasis. Some animals undergo a stress-resistant diapause stage to withstand harsh environmental conditions encountered during development. MicroRNAs are one mechanism for regulating gene expression during and after diapause. MicroRNAs downregulate target genes posttranscriptionally through the activity of the microRNA-induced silencing complex. Argonaute is the core microRNA-induced silencing complex protein that binds to both the microRNA and to other microRNA-induced silencing complex proteins. The 2 major microRNA Argonautes in the *Caenorhabditis elegans* soma are ALG-1 and ALG-2, which function partially redundantly. Loss of *alg-1* [*alg-1(0)*] causes penetrant developmental phenotypes including vulval defects and the reiteration of larval cell programs in hypodermal cells. However, these phenotypes are essentially absent if *alg-1(0)* animals undergo a diapause stage called dauer. Levels of the relevant microRNAs are not higher during or after dauer, suggesting that activity of the microRNA-induced silencing complex may be enhanced in this context. To identify genes that are required for *alg-1(0)* mutants to develop without vulval defects after dauer, we performed an RNAi screen of genes encoding conserved kinases. We focused on kinases because of their known role in modulating microRNA-induced silencing complex activity. We found RNAi knockdown of 4 kinase-encoding genes, *air-2*, *bub-1*, *chk-1*, and *nekl-3*, caused vulval defects and reiterative phenotypes in *alg-1(0)* mutants after dauer, and that these defects were more penetrant in an *alg-1(0)* background than in wild type. Our results implicate these kinases as potential regulators of microRNA-induced silencing complex activity during postdauer development in *C. elegans*.

## Introduction

The regulation of gene expression in response to changing environments is critical to maintaining homeostasis. One mechanism for modulating gene expression in different contexts is microRNA (miRNA)-mediated gene silencing. miRNAs are ∼22 nucleotide noncoding RNAs that regulate mRNA transcripts posttranscriptionally via the miRNA-induced silencing complex (miRISC). miRNAs bind to the 3′-UTR of the target mRNA via imperfect complementarity, thereby recruiting miRISC. Argonaute is the core miRNA-interacting protein that in turn binds with other protein cofactors (Bartel 2018). The protein factors in miRISC inhibit translation and cause destabilization of the transcript, thus silencing the expression of the target (Jonas and Izaurralde 2015; Bartel 2018).

Changes in miRNA activity can influence gene expression in a variety of physiological or environmental contexts (Leung and Sharp 2010; Galagali and Kim 2020). When confronted with adverse environments, some animals can enter a stress-resistant and developmentally arrested diapause stage (Hand *et al*. 2016). miRNAs have been implicated in the regulation of diapause in diverse animal species, including insects, killifish, and nematodes (Alvarez-Saavedra and Horvitz 2010; Karp *et al*. 2011; Meuti *et al*. 2018; Reynolds 2019). Although changes in miRNA levels have been documented before, during, and after diapause, little is known about changes in miRISC activity and how they may impact development in the diapause life history.

*Caenorhabditis elegans* larval development is a useful model system to explore the modulation of miRISC activity during diapause. *Caenorhabditis elegans* larvae develop through 4 larval stages (L1–L4) before becoming adults (Byerly *et al*. 1976). Larval development occurs continuously when the environment is favorable for reproduction. In unfavorable environments, larvae can pause their development after the second larval molt in the stress-resistant dauer diapause stage (Cassada and Russell 1975). If dauer larvae encounter favorable environmental conditions, they can recover and resume development through postdauer larval stages that are developmentally equivalent to those occurring in continuously developing larvae (Liu and Ambros 1991; Euling and Ambros 1996).

One developmental system regulated by miRNAs is the hypodermal stem cell–like seam cells. Seam cells divide in a particular pattern and sequence at each larval stage. At adulthood, seam cells cease dividing and differentiate (Sulston and Horvitz 1977; Ambros and Horvitz 1984). Larval vs adult seam programs are regulated by heterochronic genes that encode miRNAs and their targets (Ambros and Horvitz 1984; Rougvie and Moss 2013; Galagali and Kim 2020). The loss of heterochronic miRNAs or impaired function of miRISC proteins causes a reiterative phenotype whereby the pattern and sequence of seam cell divisions appropriate to earlier developmental stages are reiterated in later stages (Ambros and Horvitz 1984; Reinhart *et al*. 2000; Grishok *et al*. 2001; Abbott *et al*. 2005).

ALG-1 and ALG-2 are the best characterized somatic miRNA-specific Argonaute proteins in *C. elegans* and act partially redundantly (Grishok *et al*. 2001; Tops *et al*. 2006; Vasquez-Rifo *et al*. 2012). In addition, other Argonautes including ALG-5 and RDE-1 can also bind miRNAs (Seroussi *et al*. 2023). While loss of both *alg-1* and *alg-2* results in embryonic lethality, loss of *alg-1* alone [*alg-1(0)*] causes a reiterative phenotype, suggesting ALG-2 and the other Argonautes are not sufficient to mediate the activity of the heterochronic miRNAs (Grishok *et al*. 2001; Zinovyeva *et al*. 2014). Strikingly, this phenotype is strongly suppressed in postdauer *alg-1(0)* mutant animals (Karp and Ambros 2012). This finding suggests that miRNA activity is enhanced after dauer, such that the activity of *alg-2* and the remaining Argonautes becomes sufficient. Prior work has demonstrated that levels of heterochronic miRNAs are the same or reduced in postdauer larvae compared to continuously developing larvae, suggesting that the enhanced miRNA activity may arise from modulation of miRISC (Karp *et al*. 2011; Karp and Ambros 2012).

Kinases are good candidates to mediate miRISC enhancement after dauer because they are known modulators of miRISC activity. In *C. elegans* and other animals, kinases phosphorylate Argonaute and other miRISC components, thereby altering miRISC activity (Wilczynska and Bushell 2015; Frédérick and Simard 2022). To identify conserved kinases that may enhance miRNA activity after dauer, we carried out an RNAi screen for conserved kinase-encoding genes that are necessary for the postdauer suppression of *alg-1(0)* reiterative phenotypes. We found that *air-2*, *bub-1*, *chk-1*, and *nekl-3* are required to prevent vulval defects and reiterative phenotypes in *alg-1(0)* mutants after dauer. Furthermore, RNAi of these genes causes more penetrant phenotypes in an *alg-1* background than in wild type, suggesting that the kinases they encode may enhance miRNA function after dauer.

## Methods

### *Caenorhabditis elegans* strain and maintenance

All strains were maintained on Nematode Growth Medium (NGM) plates seeded with *Escherichia coli* strain OP50 at 15 or 20°C (Brenner 1974). Strains used in the study were as follows: XV88*daf-7(e1372); maIs105[col-19p::gfp]; alg-1(gk214),*VT1777*daf-7(e1372); maIs105,*GS5217*maIs105[col-19p::gfp]; alg-1(gk214),*XV85*alg-2(ok302); daf-7(e1372); maIs105;* and VT1274*alg-2(ok302); maIs105*.

### RNAi

A list of genes encoding kinases conserved between *C. elegans* and humans was obtained from Deng *et al*. (2019). Their list was generated using the OrthoList tool (Shaye and Greenwald 2011; Kim *et al*. 2018). A complete list of RNAi clones used and their sources is provided in Supplementary Table 1. RNAi bacteria were grown in LB media containing 50 µL/mL carbenicillin shaking overnight at 37°C. RNAi bacteria were then seeded on NGM plates containing 200 µg/mL IPTG and 50 µg/mL carbenicillin (“RNAi plates”). For the primary screen, 12-well plates were used, and for all other experiments, 60 mm plates were used. The primary screen was conducted in duplicate. RNAi clones that caused reiterative alae defects in postdauer adults were verified by Sanger sequencing using the M13F primer (Eurofins Genomics).

### Embryo isolation

Gravid adult hermaphrodites were treated with a bleach solution (0.4% sodium hypochlorite dissolved in 1 M NaOH) for two 2-min incubations at room temperature. The embryos were washed with sterile water and then added to RNAi plates.

### Dauer induction

The *daf-7(e1372)* allele was used to induce dauer formation (Vowels and Thomas 1992; Karp 2018). Embryos were incubated on RNAi plates at 24°C for 48–50 h, corresponding to the time just after the molt into dauer (Karp 2018).

### Dauer recovery

For the primary screen, dauer recovery was induced by washing dauer larvae off the RNAi plates with sterile water, adding the dauer larvae to fresh RNAi plates, and shifting the worms to 20°C. For all other experiments, dauer larvae were selected by incubating worms with 1% (w/v) SDS for 30 min at room temperature (Cassada and Russell 1975; Karp 2018). The dauer larvae were then washed with sterile water, added to fresh RNAi plates, and shifted to 20°C to induce dauer recovery. Postdauer larvae were incubated at 20°C for 48 h to obtain young postdauer adults.

### New RNAi clones

RNAi clones for *air-2*, *chk-1*, and *bub-1* were created based on the protocol described in Kamath and Ahringer (2003). Briefly, primers were designed to amplify regions of these genes that were distinct from those contained in the Ahringer RNAi clones. Sequences were obtained from WormBase (WS287) (Davis *et al.* 2022). The amplified sequences were cloned into the L4440 vector by TA cloning. The resulting plasmids were sequenced and then transformed into HT115 bacteria. Primers used for cloning are listed below.

*air-2* forward: 5′-TACTCCACAGAAGGGAGGGT-3′

*air-2* reverse: 5′-ATGTTGGCCACTAAGCTGAAATC-3′

*chk-1* forward: 5′-GGCGGAGAGACAGAATGCTT-3′

*chk-1* reverse: 5′-CCGAGTGCTCCACATTGACT-3′

*bub-1* forward: 5′ CCGTCGACATGTGGTCTTGA-3′

*bub-1* reverse: 5′-GAGGTTTGCGTCACTGGAGA-3′

### Microscopy

For the primary screen, bursting and/or protrusion of the vulva (Rup or Pvl phenotypes) of the postdauer adults were assessed using a dissecting microscope (Zeiss Stereo V12 fitted with M2 Bio for fluorescence). In the secondary screen, the *alg-1(0)* mutant phenotypes were assessed using a Zeiss Axio Imager D2 compound microscope. Postdauer animals were immobilized using 0.1 M levamisole on 2% agarose pads. DIC and fluorescence images were taken using an AxioCam MRm Rev 3 camera and ZEN 3.2 software. GFP was visualized with a high-efficiency GFP shift-free filter at 63× with an exposure time of 9 ms.

## Results and discussion

### RNAi screen of the conserved kinome to identify miRISC regulators acting after dauer

If *C. elegans* larvae lacking *alg-1* develop continuously from embryo to adult, the adults display reiterative phenotypes (Grishok *et al*. 2001; Zinovyeva *et al*. 2014). In contrast, these phenotypes are suppressed if *alg-1(0)* mutant larvae develop through the dauer diapause stage (Karp and Ambros 2012). We hypothesized that modulation of miRISC activity in postdauer animals could account for this difference. Since kinases are known to modulate miRISC in various physiological and environmental contexts (Wu *et al*. 2011; Alessi *et al*. 2015; Olejniczak *et al*. 2018), we designed an RNAi screen of all of the conserved kinases in *C. elegans* (Kim *et al*. 2018; Deng *et al*. 2019). If postdauer *alg-1(0)* mutants display reiterative phenotypes after depletion of a particular kinase, that kinase is a candidate for modulating miRISC function in postdauer animals.

**Fig. 1.**
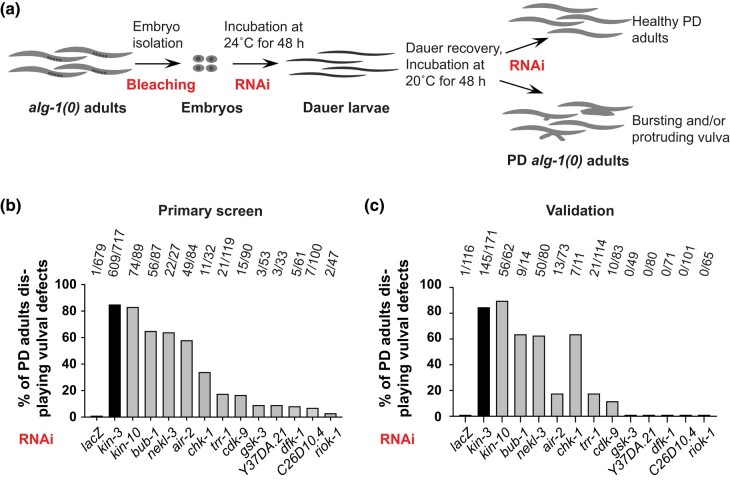
Kinases identified in a primary screen for enhancement of an *alg-1(0)* phenotype after dauer. a) Strategy for RNAi screening. The full genotype of the strain used for screening was *daf-7(e1372); maIs105[col-19p::gfp]; alg-1(gk214)*. The *daf-7(e1372)* allele was used to induce dauer formation at 24°C and allow recovery at lower temperatures (see *Methods* for details). b) RNAi clones that produced vulval defects (Rup and/or Pvl phenotypes) in the primary screen. The screen was performed in duplicate, and clones that produced vulval defects in both plates are shown here. Supplementary Table 1 shows data for all clones that produced vulval defects in at least 1 well. The remaining clones produced no vulval defects in either well (≥20 adults scored per well). Numbers in b) and c) indicate the number of postdauer (PD) adults displaying vulval defects over the total PD adults scored. c) The 12 hits from the primary screen were retested for vulval defects. Seven genes caused bursting and/or protrusion of the vulva in PD *alg-1(0)* adults. Among these 7 genes, 6 were novel *alg-1* interactors.

To perform our RNAi screen, we used an *alg-1(0)* strain that contained the temperature-sensitive *daf-7(e1372)* allele to induce dauer formation at 24°C and allow recovery at lower temperatures (Vowels and Thomas 1992; Karp 2018). A *col-19p::gfp* transgene was also included to allow analysis of that aspect of adult cell fate (Liu *et al.* 1995). Moving forward, unless otherwise specified, it should be presumed that all strains described here contain *daf-7(e1372); maIs105[col-19p::gfp]* in the background. Embryos were added to RNAi plates and incubated at 24°C for 2 days or until just after dauer formation. Dauer larvae were washed off with water, transferred to fresh RNAi plates, and shifted to 20°C to promote recovery. Postdauer *alg-1(0)* adults were then screened for the vulval defects associated with compromised miRISC function (Fig. 1a). We used *lacZ* RNAi as a negative control and *kin-3* RNAi as a positive control because we previously showed that RNAi of *kin-3* caused reiterative phenotypes in postdauer *alg-1(0)* mutants (Alessi *et al*. 2015). *kin-3* encodes the catalytic subunit of casein kinase 2 (CK2), and CK2 phosphorylates the miRISC proteins ALG-1 and CGH-1 to promote miRISC function (Alessi *et al*. 2015; Shah *et al*. 2023). We used a previously published list of the 247 *C. elegans* kinases with human orthologs (Kim *et al*. 2018; Deng *et al*. 2019). Some kinases are represented by more than 1 RNAi clone, giving us 286 RNAi clones in total (Supplementary Table 1).

In our primary screen, performed in duplicate, we used a dissecting microscope to look for vulval defects in *alg-1(0)* postdauer adults. Vulval defects included both bursting (Rup) and protruding vulva (Pvl) phenotypes. We focused on vulval defects because these phenotypes are easily visible in the dissecting microscope. As expected, we saw almost no vulval defects after treatment with *lacZ* RNAi (Fig. 1b). In contrast, *kin-3* RNAi caused a high penetrance of vulval defects (Fig. 1b). We found 12 additional conserved kinases that caused similar phenotypes. We observed a broad range of penetrance of vulval defects that could be due to different degrees of requirement for vulval development and/or variable effectiveness of RNAi.

To ensure our results were reproducible, we retested the 12 hits from the primary screen. In addition, in this validation experiment, we selected dauer larvae by treatment with 1% SDS (Cassada and Russell 1975). Treatment with SDS ensured that the adults we screened were indeed postdauer and not displaying vulval defects because the larvae had escaped dauer formation. Upon retesting, 7 genes displayed vulval defects (Fig. 1c). One of these genes, *kin-10*, encodes the regulatory subunit of the of CK2 protein and was therefore expected (Alessi *et al*. 2015; Shah *et al*. 2023). We therefore focused on the remaining 6 kinase-encoding genes.

### Five kinase-encoding genes contribute to adult alae formation in *alg-1(0)* postdauer adults

In addition to vulval defects, *alg-1(0)* mutant adults display gapped or missing alae after continuous development (Grishok *et al*. 2001; Vasquez-Rifo *et al*. 2012). This reiterative phenotype occurs in heterochronic mutants because the underlying seam cells repeat larval cell programs (Ambros and Horvitz 1984). In addition to gapped or missing alae, *alg-1(0)* animals that develop continuously can display other defects in alae formation, including small breaks and growth that deviates from the straight line observed in wild-type adults (Alessi *et al*. 2015; Shah *et al*. 2023). Furthermore, the loss of *alg-1* can reduce the expression of the adult cell fate marker *col-19p::gfp*, albeit at low penetrance (Zinovyeva *et al*. 2015). If the kinases we identified were important for miRNA function, we would expect depletion of these genes to cause alae defects and possibly reduced *col-19p::gfp* in the hypodermis of postdauer *alg-1(0)* adults.

**Fig. 2.**
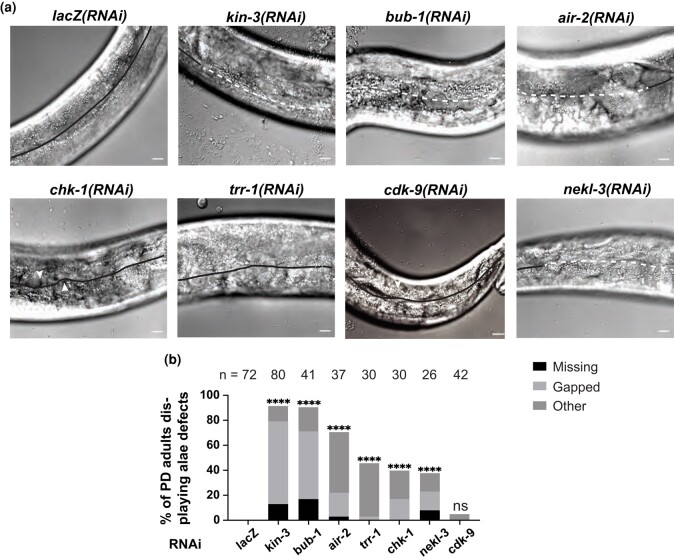
RNAi of 5 kinase-encoding genes produces defects in adult alae formation in postdauer *alg-1(0)* mutants. a) Comparison of alae phenotypes in postdauer *daf-7(e1372); col-19p::gfp; alg-1(0)* adults. A compound microscope with DIC optics was used to visualize the presence of alae in postdauer adult animals. Solid lines represent complete alae, dashed lines represent gapped alae, and arrowheads show other alae defects including small breaks or curved or swirly alae. Scale bars = 10 µm. b) Quantification of alae defects. The number of adults scored (*n*) for each RNAi condition is listed. **** = *P* < 0.0002; ns = *P* > 0.05, Fisher exact test.

To test the hypothesis that these kinases are required to prevent reiterative phenotypes in postdauer *alg-1(0)* adults, we performed RNAi on the 6 hits and looked at adult alae formation and *col-19p::gfp* expression with DIC and fluorescence microscopy. We found that knocking down 5 of the 6 genes with RNAi resulted in penetrant alae defects (Fig. 2). The most penetrant alae phenotypes were caused by *bub-1* RNAi. RNAi of *cdk-9* produced no reiterative phenotypes and occasional other alae defects. While *lacZ* RNAi produced no alae defects at all, *cdk-9* RNAi was not significantly different from the *lacZ* control. In contrast to the alae defects produced by RNAi, *col-19p::gfp* expression was not reduced by RNAi of any of the 6 candidate genes and only moderately downregulated in our *kin-3* positive control (Supplementary Fig. 1). Uncoupling of alae defects and *col-19p::gfp* expression has been previously observed in some mutant backgrounds (Hada *et al*. 2010; Hansen *et al*. 2022).

### RNAi of some kinases produces a stronger phenotype in a miRISC-sensitized background and during postdauer development

**Fig. 3.**
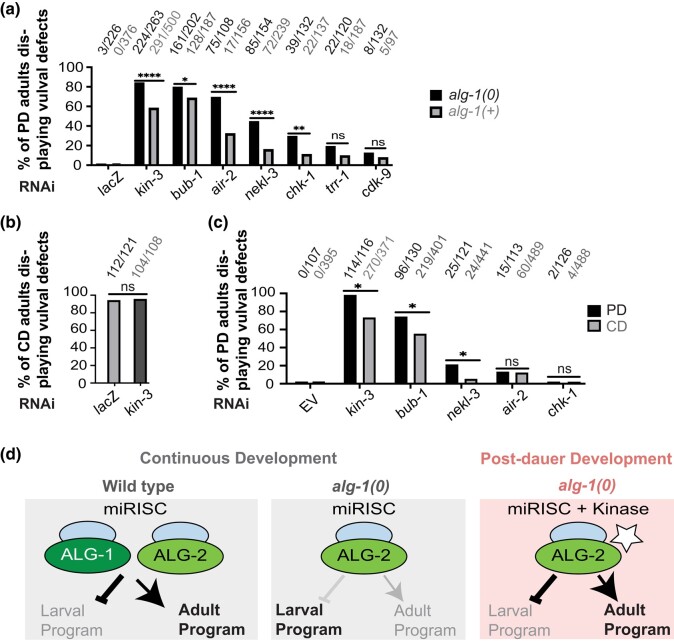
RNAi of some kinases caused more penetrant phenotypes after dauer and when miRISC was compromised. a) Vulval defects in postdauer adults with wild-type miRISC were compared to animals with compromised miRISC function [*alg-1(0)* mutants]. Both strains contained *daf-7(e1372); col-19p::gfp* in the background. b) During continuous development, the penetrance of vulval defects in *alg-1(0)* adults is high such that no further enhancement is seen when *kin-3* is knocked down. c) Vulval defects in *alg-2(0)* young adults after dauer (PD, postdauer) or after continuous development (CD). In b) and c), dauer was induced by *daf-7(e1372)*, whereas continuously developing animals were wild type for *daf-7*. *P* > 0.05 (ns), <0.0332 (*), <0.0021 (**), <0.0002 (****), Fisher exact test. Empty vector (EV) was used as a negative control. d) Model for role of miRISC activity in different developmental contexts to control larval vs adult seam cell developmental programs. Note that other Argonautes could act in addition to or in place of ALG-2.

Vulval defects can be caused by the depletion of genes that regulate processes other than miRISC activity. For example, loss of genes involved in regulation of uterine development can produce similar phenotypes (Seydoux and Greenwald 1989). If the kinases identified in our screen enhance miRISC activity after dauer, we would expect that RNAi of these kinases would produce a stronger phenotype in *alg-1(0)* adults than when *alg-1* was wild type. To test this hypothesis, we performed RNAi on *alg-1(0)* and *alg-1(+)* animals in parallel. RNAi of *bub-1*, *air-2*, *nekl-3*, and *chk-1* each displayed a more penetrant phenotype in the miRISC-compromised background (Fig. 3a). RNAi of *trr-1* or *cdk-9* caused a low-penetrance phenotype in both backgrounds, and there was no significant difference between the backgrounds (Fig. 3a). While these data do not rule out the involvement of any of these genes in regulating miRISC activity, they provide higher confidence in *bub-1*, *air-2*, *nekl-3*, and *chk-1* than *trr-1* or *cdk-9*.

Since *alg-1(0)* mutants display vulval defects during continuous development but not after dauer, we next wondered whether the kinases we identified play a greater role in preventing vulval defects in postdauer hermaphrodites than in those that develop continuously, particularly when miRISC is compromised. We were unable to test this question using *alg-1(0)* mutants because they display penetrant phenotypes during continuous development (Fig. 3b). However, animals lacking *alg-2* appear superficially wild type in both life histories (Grishok *et al*. 2001; Karp and Ambros 2012). We therefore compared phenotypes in each life history following RNAi of *bub-1*, *nekl-3*, *air-2*, and *chk-1* in an *alg-2(0)* mutant background. We found that RNAi of *bub-1* and *nekl-3* both produced statistically more penetrant phenotypes after dauer (Fig. 3c), consistent with these kinases playing a larger role in postdauer development.

In contrast, RNAi of *air-2* and *chk-1* produced low-penetrance phenotypes in both life histories in the *alg-2(0)* background (Fig. 3c). While in this latter case there was no statistical difference between postdauer and continuously developing animals, observing a decrease in penetrance in adults that had developed continuously would be difficult given the low-penetrance phenotype in postdauer adults. Thus, we are unable to draw strong conclusions about the relative role of *air-2* and *chk-1* in postdauer vs continuous development.

Finally, we performed RNAi of *bub-1*, *air-2*, *nekl-3*, and *chk-1* using independent clones that target different regions of each gene than the region targeted in the original clones. We observed vulval defects using each set of clones, albeit at reduced penetrance (Supplementary Fig. 2). The percent of vulval defects observed for each clone was statistically different from the empty vector control, except the newly created *chk-1* clone (*P* = 0.088). Both original and newly created *chk-1* clones produced low-penetrance phenotypes, whereas the negative control produced no vulval defects out of 226 adults examined.

### Kinases identified in this screen

We have identified 4 kinases that, when depleted, reproducibly enhanced 2 different *alg-1(0)* phenotypes in postdauer animals and whose depletion caused more severe defects in a miRISC-compromised background than in wild type (Table 1). The known roles for these kinases are described below.

**Table 1.** Conservation and function of genes isolated in the screen.

*C. elegans* gene	Human ortholog	Connection to microRNA function	Characterized function	References
*air-2*	AURKB (Aurora kinase B)	None	Chromosome segregation and cytokinesis in mitosis and meiosis	Schumacher *et al*. (1998); Rogers *et al*. (2002)
*bub-1*	BUB1 (BUB1 mitotic checkpoint serine/threonine kinase)	None	Chromosome alignment and segregation, spindle assembly, and promotion of anaphase in mitosis and meiosis	Sharp-Baker and Chen (2001); Kim *et al*. (2015); Edwards *et al*. (2018)
*chk-1*	CHEK1 (checkpoint kinase 1)	None	DNA damage checkpoint and embryonic S-M checkpoint kinase	Kalogeropoulos *et al*. (2004)
*nekl-3*	NEK6/7 (NIMA-related kinase 6/7)	Identified as a weak enhancer of the *let-7(mg279)* Rup phenotype during continuous development	Clathrin-mediated endocytosis of cargo necessary for molting	Parry *et al*. (2007); Joseph *et al*. (2020)

Among the 4 genes identified as the highest confidence candidates in our screen, *nekl-3* is the only one to have been previously implicated in the regulation of miRNA activity. *nekl-3* was isolated in a large-scale RNAi screen for genes that enhance the Rup phenotype of mutants with reduced activity of the *let-7* miRNA (Parry *et al*. 2007). Although *nekl-3* was a relatively weak enhancer and the screen was performed during continuous (nondauer) development, this observation is consistent with the hypothesis that *nekl-3* can promote miRISC function. This role for *nekl-3* remains unexplored. In contrast, a role for *nekl-3* in the regulation of molting has been well characterized. *nekl-3* is required for the internalization via clathrin-mediated endocytosis of cargo important for molting (Yochem *et al*. 2015; Joseph *et al*. 2020). Specifically, *nekl-*3 is required for the L1–L2 and L2–L3 molts but not later molts during continuous development. In the dauer life history, *nekl-*3 has a reduced requirement for the L2d-dauer molt and is dispensable for postdauer molting (Binti *et al*. 2022).

In mammals, the *nekl-3* orthologs, NEK family proteins, are best characterized for their role in mitosis and chromosome segregation (Fry *et al*. 2012). Notably, *air-2* and *bub-1* both encode conserved proteins that are critical for chromosome segregation and mitosis and meiosis across species (Schumacher *et al*. 1998; Sharp-Baker and Chen 2001; Rogers *et al*. 2002; Kim *et al*. 2015). Neither of these genes has been previously implicated in miRNA activity. Previous large-scale screens conducted during continuous development found that RNAi knockdown of *bub-1* produced Pvl and bursting phenotypes (Fraser *et al*. 2000). Loss-of-function *bub-1* mutants display a vulvaless (Vul) phenotype and a low-penetrance Pvl phenotype (Wang *et al*. 2009). However, whether these phenotypes are related to the characterized role in chromosome segregation or whether they may indicate a role for *bub-1* in regulating miRNA activity is unknown. Finally, *chk-1* encodes a serine–threonine kinase that plays a role in the checkpoint response to DNA damage and in the S-M checkpoint in embryos (Kalogeropoulos *et al*. 2004).

If the kinases we identified do regulate miRISC, this regulation could be direct or indirect. Consistent with direct regulation, *bub-1* and *nekl-3* are expressed in tissues displaying *alg-1(0)* phenotypes, including the vulva and the hypodermis (Tarailo-Graovac *et al*. 2010; Yochem *et al*. 2015). Postembryonic expression data outside of the germline have not been described for *air-2* or *chk-1*. Furthermore, no expression data for any of the 4 kinases have been reported in postdauer animals. It will be interesting to determine whether these kinases are expressed in relevant tissues in that context. In addition, core miRISC proteins including ALG-1, ALG-2, AIN-1, and AIN-2 have many phosphosites identified by phosphoproteomic studies (Huang *et al*. 2020; Li *et al*. 2021). It is unclear whether these sites may be phosphorylated by the kinases identified in this study. There are no defined consensus sequences phosphorylated by BUB-1 and NEKL-3. There are loose consensus sequences defined for the human orthologs of AIR-2 ([RK]X[TS]) and CHK-1 (RXX[TS]) that may also apply to *C. elegans* (Sanchez *et al*. 1997; Cheeseman *et al*. 2002; Chen *et al*. 2003; Bishop *et al*. 2005; Ferrandiz *et al*. 2018). ALG-1, ALG-2, AIN-1, and AIN-2 all contain sequences that match these loose consensus sites; however, these sequences were not found to be phosphorylated in the aforementioned phosphoproteomic studies (Huang *et al*. 2020; Li *et al*. 2021). In addition, these studies were not performed in postdauer animals. It will be interesting to explore the possibility of direct regulation of miRISC components by these kinases in future work.

## Conclusion

The goal of this study was to identify kinase-encoding genes that may enhance miRISC function in postdauer development. This study initially found 6 candidate genes required to prevent developmental phenotypes in postdauer *alg-1(0)* animals. Four of these genes, *bub-1*, *air-2*, *nekl-3*, and *chk-1*, showed higher penetrance phenotypes in a miRISC-compromised background, making these 4 genes the most likely to affect miRISC activity after dauer. *bub-1* and *nekl-3* produced more penetrant phenotypes after dauer than during continuous development in an *alg-2(0)* background, whereas *air-2* and *chk-1* produced low-penetrance phenotypes in both life histories in an *alg-2(0)* background.

Prior to this work, the only kinase-encoding gene known to enhance *alg-1(0)* phenotypes after dauer was *kin-3*, used as a positive control in this study (Alessi *et al*. 2015). During continuous development, 3 kinases have been shown to phosphorylate miRISC components to modulate different aspects of miRNA activity: KIN-1/PKA, casein kinase 1 (CK1), and CK2 (Alessi *et al*. 2015; Quévillon Huberdeau *et al*. 2022; Shah *et al*. 2023). In our screen, only the CK2-encoding genes *kin-3* and *kin-10* were found to be necessary to prevent vulval defects in *alg-1(0)* mutants after dauer, whereas RNAi of *kin-1* and *kin-19*, encoding PKA and CK1, respectively, did not cause vulval defects in our primary screen (*kin-1*, 0/40; *kin-19*, 0/60). While these findings may be explained by incomplete knockdown of *kin-1* and *kin-19*, it is also possible that distinct kinases modulate miRISC in the dauer life history. This study lays the foundation for future studies that can dissect the molecular relationship between these genes and miRISC in *C. elegans* and other species.

## Supplementary Material

jkae007_Supplementary_Data

## Data Availability

All *C. elegans* strains and newly made RNAi clones are available upon request. The authors state that all data necessary for confirming the conclusions of the article are present within the article, figures, and tables, including supplementary information. Supplemental material available at G3 online.

## References

[jkae007-B1] Abbott AL , Alvarez-Saavedra E, Miska EA, Lau NC, Bartel DP, Horvitz HR, Ambros V. 2005. The *let-7* microRNA family members *mir-48, mir-84*, and *mir-241* function together to regulate developmental timing in *Caenorhabditis elegans*. Dev Cell. 9(3):403–414. doi:10.1016/j.devcel.2005.07.009. 16139228 PMC3969732

[jkae007-B2] Alessi AF , Khivansara V, Han T, Freeberg MA, Moresco JJ, Tu PG, Montoye E, Yates JR 3rd, Karp X, Kim JK. 2015. *Casein kinase II* promotes target silencing by miRISC through direct phosphorylation of the DEAD-box RNA helicase CGH-1. Proc Natl Acad Sci U S A. 112(52):E7213–E7222. doi:10.1073/pnas.1509499112. 26669440 PMC4702986

[jkae007-B3] Alvarez-Saavedra E , Horvitz HR. 2010. Many families of *C. elegans* microRNAs are not essential for development or viability. Curr Biol. 20(4):367–373. doi:10.1016/j.cub.2009.12.051. 20096582 PMC2844791

[jkae007-B4] Ambros V , Horvitz HR. 1984. Heterochronic mutants of the nematode *Caenorhabditis elegans*. Science 226(4673):409–416. doi:10.1126/science.6494891. 6494891

[jkae007-B5] Bartel DP . 2018. Metazoan MicroRNAs. Cell. 173(1):20–51. doi:10.1016/j.cell.2018.03.006. 29570994 PMC6091663

[jkae007-B6] Binti S , Melinda RV, Joseph BB, Edeen PT, Miller SD, Fay DS. 2022. A life cycle alteration can correct molting defects in *Caenorhabditis elegans*. Dev Biol. 483:143–156. doi:10.1016/j.ydbio.2022.01.001. 35038442 PMC8867747

[jkae007-B7] Bishop JD , Han Z, Schumacher JM. 2005. The *Caenorhabditis elegans* aurora B kinase AIR-2 phosphorylates and is required for the localization of a BimC kinesin to meiotic and mitotic spindles. Mol Biol Cell. 16(2):742–756. doi:10.1091/mbc.e04-08-0682. 15548597 PMC545908

[jkae007-B8] Brenner S . 1974. The genetics of *Caenorhabditis elegans*. Genetics 77(1):71–94. doi:10.1093/genetics/77.1.71. 4366476 PMC1213120

[jkae007-B9] Byerly L , Cassada RC, Russell RL. 1976. The life cycle of the nematode *Caenorhabditis elegans*. I. Wild-type growth and reproduction. Dev Biol. 51(1):23–33. doi:10.1016/0012-1606(76)90119-6. 988845

[jkae007-B10] Cassada RC , Russell RL. 1975. The dauerlarva, a post-embryonic developmental variant of the nematode *Caenorhabditis elegans*. Dev Biol. 46(2):326–342. doi:10.1016/0012-1606(75)90109-8. 1183723

[jkae007-B11] Cheeseman IM , Anderson S, Jwa M, Green EM, Kang J, Yates JR 3rd, Chan CS, Drubin DG, Barnes G. 2002. Phospho-regulation of kinetochore-microtubule attachments by the Aurora kinase Ipl1p. Cell 111(2):163–172. doi:10.1016/S0092-8674(02)00973-X. 12408861

[jkae007-B12] Chen M-S , Ryan CE, Piwnica-Worms H. 2003. Chk1 kinase negatively regulates mitotic function of Cdc25A phosphatase through 14-3-3 binding. Mol Cell Biol. 23(21):7488–7497. doi:10.1128/MCB.23.21.7488-7497.2003. 14559997 PMC207598

[jkae007-B13] Davis P , Zarowiecki M, Arnaboldi V, Becerra A, Cain S, Chan J, Chen WJ, Cho J, da Veiga, Diamantakis S. 2022. WormBase in 2022-data, processes, and tools for analyzing *Caenorhabditis elegans*. Genetics 220(4):iyac003. doi:10.1093/genetics/iyac003. PMC8982018 35134929

[jkae007-B14] Deng Y , Luo KL, Shaye DD, Greenwald I. 2019. A screen of the conserved kinome for negative regulators of LIN-12 negative regulatory region (“NRR”)-missense activity in *Caenorhabditis elegans*. G3 (Bethesda). 9(11):3567–3574. doi:10.1534/g3.119.400471. 31519743 PMC6829150

[jkae007-B15] Edwards F , Maton G, Gareil N, Canman JC, Dumont J. 2018. BUB-1 promotes amphitelic chromosome biorientation via multiple activities at the kinetochore. Elife 7:e40690. doi:10.7554/eLife.40690. 30547880 PMC6303103

[jkae007-B16] Euling S , Ambros V. 1996. Reversal of cell fate determination in *Caenorhabditis elegans* vulval development. Development 122(8):2507–2515. doi:10.1242/dev.122.8.2507. 8756295

[jkae007-B17] Ferrandiz N , Barroso C, Telecan O, Shao N, Kim HM, Testori S, Faull P, Cutillas P, Snijders AP, Colaiácovo MP, et al 2018. Spatiotemporal regulation of Aurora B recruitment ensures release of cohesion during *C. elegans* oocyte meiosis. Nat Commun. 9(1):834. doi:10.1038/s41467-018-03229-5. 29483514 PMC5827026

[jkae007-B18] Fraser AG , Kamath RS, Zipperlen P, Martinez-Campos M, Sohrmann M, Ahringer J. 2000. Functional genomic analysis of *C. elegans* chromosome I by systematic RNA interference. Nature 408(6810):325–330. doi:10.1038/35042517. 11099033

[jkae007-B19] Frédérick P , Simard MJ. 2022. Regulation and different functions of the animal microRNA-induced silencing complex. Wiley Interdiscip Rev RNA. 13(4):e1701. doi:10.1002/wrna.1701. 34725940

[jkae007-B20] Fry AM , O’Regan L, Sabir SR, Bayliss R. 2012. Cell cycle regulation by the NEK family of protein kinases. J Cell Sci. 125(Pt\ 19):4423–4433. doi:10.1242/jcs.111195. 23132929 PMC3500863

[jkae007-B21] Galagali H , Kim JK. 2020. The multifaceted roles of microRNAs in differentiation. Curr Opin Cell Biol. 67:118–140. doi:10.1016/j.ceb.2020.08.015. 33152557 PMC8607530

[jkae007-B22] Grishok A , Pasquinelli AE, Conte D, Li N, Parrish S, Ha I, Baillie DL, Fire A, Ruvkun G, Mello CC. 2001. Genes and mechanisms related to RNA interference regulate expression of the small temporal RNAs that control *C. elegans* developmental timing. Cell 106(1):23–34. doi:10.1016/S0092-8674(01)00431-7. 11461699

[jkae007-B23] Hada K , Asahina M, Hasegawa H, Kanaho Y, Slack FJ, Niwa R. 2010. The nuclear receptor gene nhr-25 plays multiple roles in the *Caenorhabditis elegans* heterochronic gene network to control the larva-to-adult transition. Dev Biol. 344(2):1100–1109. doi:10.1016/j.ydbio.2010.05.508. 20678979 PMC2915939

[jkae007-B24] Hand SC , Denlinger DL, Podrabsky JE, Roy R. 2016. Mechanisms of animal diapause: recent developments from nematodes, crustaceans, insects, and fish. Am J Physiol. 310(11):R1193–R1211. doi:10.1152/ajpregu.00250.2015. PMC4935499 27053646

[jkae007-B25] Hansen MA , Dahal A, Bernstein TA, Kohtz C, Ali S, Daul AL, Montoye E, Panzade GP, Alessi AF, Flibotte S , et al ztf-16 is a novel heterochronic modulator that opposes adult cell fate in dauer and continuous life histories in *Caenorhabditis elegans*. bioRxiv 496913, 10.1101/2022.06.20.496913, preprint: not peer reviewed.

[jkae007-B26] Huang J , Wu Z, Zhang X. 2020. Short-term mild temperature-stress-induced alterations in the *C. elegans* phosphoproteome. Int J Mol Sci. 21(17):6409. doi:10.3390/ijms21176409. 32899194 PMC7504583

[jkae007-B27] Jonas S , Izaurralde E. 2015. Towards a molecular understanding of microRNA-mediated gene silencing. Nat Rev Genet. 16(7):421–433. doi:10.1038/nrg3965. 26077373

[jkae007-B28] Joseph BB , Wang Y, Edeen P, Lažetić V, Grant BD, Fay DS. 2020. Control of clathrin-mediated endocytosis by NIMA family kinases. PLoS Genet. 16(2):e1008633. doi:10.1371/journal.pgen.1008633. 32069276 PMC7048319

[jkae007-B29] Kalogeropoulos N , Christoforou C, Green AJ, Gill S, Ashcroft NR. 2004. *chk-1* is an essential gene and is required for an S-M checkpoint during early embryogenesis. Cell Cycle 3(9):1194–1198. doi:10.4161/cc.3.9.1116. 15326393

[jkae007-B30] Kamath RS , Ahringer J. 2003. Genome-wide RNAi screening in *Caenorhabditis elegans*. Methods 30(4):313–321. doi:10.1016/S1046-2023(03)00050-1. 12828945

[jkae007-B31] Karp X . 2018. Working with dauer larvae. WormBook 2018:1–19. doi:10.1895/wormbook.1.180.1. PMC5237411 27417559

[jkae007-B32] Karp X , Ambros V. 2012. Dauer larva quiescence alters the circuitry of microRNA pathways regulating cell fate progression in *C. elegans*. Development 139(12):2177–2186. doi:10.1242/dev.075986. 22619389 PMC3357911

[jkae007-B33] Karp X , Hammell M, Ow MC, Ambros V. 2011. Effect of life history on microRNA expression during *C. elegans* development. RNA 17(4):639–651. doi:10.1261/rna.2310111. 21343388 PMC3062175

[jkae007-B34] Kim T , Moyle MW, Lara-Gonzalez P, De Groot C, Oegema K, Desai A. 2015. Kinetochore-localized BUB-1/BUB-3 complex promotes anaphase onset in *C. elegans*. J Cell Biol. 209(4):507–517. doi:10.1083/jcb.201412035. 25987605 PMC4442812

[jkae007-B35] Kim W , Underwood RS, Greenwald I, Shaye DD. 2018. OrthoList 2: a new comparative genomic analysis of human and *Caenorhabditis elegans* genes. Genetics 210(2):445–461. doi:10.1534/genetics.118.301307. 30120140 PMC6216590

[jkae007-B36] Leung AKL , Sharp PA. 2010. MicroRNA functions in stress responses. Mol Cell. 40(2):205–215. doi:10.1016/j.molcel.2010.09.027. 20965416 PMC2996264

[jkae007-B37] Li WJ , Wang CW, Tao L, Yan YH, Zhang MJ, Liu ZX, Li YX, Zhao HQ, Li XM, He XD, et al 2021. Insulin signaling regulates longevity through protein phosphorylation in *Caenorhabditis elegans*. Nat Commun. 12(1):4568. doi:10.1038/s41467-021-24816-z. 34315882 PMC8316574

[jkae007-B38] Liu Z , Ambros V. 1991. Alternative temporal control systems for hypodermal cell differentiation in *Caenorhabditis elegans*. Nature 350(6314):162–165. doi:10.1038/350162a0. 26502479

[jkae007-B39] Liu Z , Kirch S, Ambros V. 1995. The *Caenorhabditis elegans* heterochronic gene pathway controls stage-specific transcription of collagen genes. Development 121(8):2471–2479. 7671811 10.1242/dev.121.8.2471

[jkae007-B40] Meuti ME , Bautista-Jimenez R, Reynolds JA. 2018. Evidence that microRNAs are part of the molecular toolkit regulating adult reproductive diapause in the mosquito, *Culex pipiens*. PLoS One 13(11):e0203015. doi:10.1371/journal.pone.0203015. 30496183 PMC6264513

[jkae007-B41] Olejniczak M , Kotowska-Zimmer A, Krzyzosiak W. 2018. Stress-induced changes in miRNA biogenesis and functioning. Cell Mol Life Sci. 75(2):177–191. doi:10.1007/s00018-017-2591-0. 28717872 PMC5756259

[jkae007-B42] Parry DH , Xu J, Ruvkun G. 2007. A whole-genome RNAi screen for *C. elegans* miRNA pathway genes. Curr Biol. 17(23):2013–2022. doi:10.1016/j.cub.2007.10.058. 18023351 PMC2211719

[jkae007-B43] Quévillon Huberdeau M , Shah VN, Nahar S, Neumeier J, Houle F, Bruckmann A, Gypas F, Nakanishi K, Großhans H, Meister G, et al 2022. A specific type of Argonaute phosphorylation regulates binding to microRNAs during *C. elegans* development. Cell Rep. 41(11):111822. doi:10.1016/j.celrep.2022.111822. 36516777 PMC10436268

[jkae007-B44] Reinhart BJ , Slack FJ, Basson M, Pasquinelli AE, Bettinger JC, Rougvie AE, Horvitz HR, Ruvkun G. 2000. The 21-nucleotide let-7 RNA regulates developmental timing in *Caenorhabditis elegans*. Nature 403(6772):901–906. doi:10.1038/35002607. 10706289

[jkae007-B45] Reynolds JA . 2019. Noncoding RNA regulation of dormant states in evolutionarily diverse animals. Biol Bull. 237(2):192–209. doi:10.1086/705484. 31714856

[jkae007-B46] Rogers E , Bishop JD, Waddle JA, Schumacher JM, Lin R. 2002. The aurora kinase AIR-2 functions in the release of chromosome cohesion in *Caenorhabditis elegans* meiosis. J Cell Biol. 157(2):219–229. doi:10.1083/jcb.200110045. 11940606 PMC1855215

[jkae007-B47] Rougvie AE , Moss EG. 2013. Developmental transitions in *C. elegans* larval stages. Curr Top Dev Biol. 105:153–180. doi:10.1016/B978-0-12-396968-2.00006-3. 23962842

[jkae007-B48] Sanchez Y , Wong C, Thoma RS, Richman R, Wu Z, Piwnica-Worms H, Elledge SJ. 1997. Conservation of the Chk1 checkpoint pathway in mammals: linkage of DNA damage to Cdk regulation through Cdc25. Science 277(5331):1497–1501. doi:10.1126/science.277.5331.1497. 9278511

[jkae007-B49] Schumacher JM , Golden A, Donovan PJ. 1998. AIR-2: an Aurora/Ipl1-related protein kinase associated with chromosomes and midbody microtubules is required for polar body extrusion and cytokinesis in *Caenorhabditis elegans* embryos. J Cell Biol. 143(6):1635–1646. doi:10.1083/jcb.143.6.1635. 9852156 PMC2132979

[jkae007-B50] Seroussi U , Lugowski A, Wadi L, Lao RX, Willis AR, Zhao W, Sundby AE, Charlesworth AG, Reinke AW, Claycomb JM. 2023. A comprehensive survey of *C. elegans* argonaute proteins reveals organism-wide gene regulatory networks and functions. Elife 12:e83853. doi:10.7554/eLife.83853. 36790166 PMC10101689

[jkae007-B51] Seydoux G , Greenwald I. 1989. Cell autonomy of lin-12 function in a cell fate decision in *C. elegans*. Cell 57(7):1237–1245. doi:10.1016/0092-8674(89)90060-3. 2736627

[jkae007-B52] Shah VN , Neumeier J, Huberdeau MQ, Zeitler DM, Bruckmann A, Meister G, Simard MJ. 2023. *Casein kinase 1* and *2* phosphorylate Argonaute proteins to regulate miRNA-mediated gene silencing. EMBO Rep. 24(11):e57250. doi:10.15252/embr.202357250. 37712432 PMC10626430

[jkae007-B53] Sharp-Baker H , Chen R-H. 2001. Spindle checkpoint protein Bub1 is required for kinetochore localization of Mad1, Mad2, Bub3, and Cenp-E, independently of its kinase activity. J Cell Biol. 153(6):1239–1250. doi:10.1083/jcb.153.6.1239. 11402067 PMC2192030

[jkae007-B54] Shaye DD , Greenwald I. 2011. OrthoList: a compendium of *C. elegans* genes with human orthologs. PLoS One 6(5):e20085. doi:10.1371/journal.pone.0020085. 21647448 PMC3102077

[jkae007-B55] Sulston JE , Horvitz HR. 1977. Post-embryonic cell lineages of the nematode, *Caenorhabditis elegans*. Dev Biol. 56(1):110–156. doi:10.1016/0012-1606(77)90158-0. 838129

[jkae007-B56] Tarailo-Graovac M , Wang J, Chu JS, Tu D, Baillie DL, Chen N. 2010. Spindle assembly checkpoint genes reveal distinct as well as overlapping expression that implicates MDF-2/Mad2 in postembryonic seam cell proliferation in *Caenorhabditis elegans*. BMC Cell Biol. 11(1):71. doi:10.1186/1471-2121-11-71. 20858267 PMC2955571

[jkae007-B57] Tops BBJ , Plasterk RHA, Ketting RF. 2006. The *Caenorhabditis elegans* Argonautes ALG-1 and ALG-2: almost identical yet different. Cold Spring Harb Symp Quant Biol. 71(0):189–194. doi:10.1101/sqb.2006.71.035. 17381296

[jkae007-B58] Vasquez-Rifo A , Jannot G, Armisen J, Labouesse M, Bukhari SI, Rondeau EL, Miska EA, Simard MJ. 2012. Developmental characterization of the microRNA-specific *C. elegans* Argonautes *alg-1* and *alg-2*. PLoS One 7(3):e33750. doi:10.1371/journal.pone.0033750. 22448270 PMC3309000

[jkae007-B59] Vowels JJ , Thomas JH. 1992. Genetic analysis of chemosensory control of dauer formation in *Caenorhabditis elegans*. Genetics 130(1):105–123. doi:10.1093/genetics/130.1.105. 1732156 PMC1204785

[jkae007-B60] Wang X , Liu M, Li W, Suh CD, Zhu Z, Jin Y, Fan Q. 2009. The function of a spindle checkpoint gene *bub-1* in *C. elegans* development. PLoS One 4(6):e5912. doi:10.1371/journal.pone.0005912. 19526056 PMC2691579

[jkae007-B61] Wilczynska A , Bushell M. 2015. The complexity of miRNA-mediated repression. Cell Death Differ. 22(1):22–33. doi:10.1038/cdd.2014.112. 25190144 PMC4262769

[jkae007-B62] Wu C , So J, Davis-Dusenbery BN, Qi HH, Bloch DB, Shi Y, Lagna G, Hata A. 2011. Hypoxia potentiates microRNA-mediated gene silencing through posttranslational modification of Argonaute2. Mol Cell Biol. 31(23):4760–4774. doi:10.1128/MCB.05776-11. 21969601 PMC3232924

[jkae007-B63] Yochem J , Lažetić V, Bell L, Chen L, Fay D. 2015. *C. elegans* NIMA-related kinases NEKL-2 and NEKL-3 are required for the completion of molting. Dev Biol. 398(2):255–266. doi:10.1016/j.ydbio.2014.12.008. 25523392 PMC4314388

[jkae007-B64] Zinovyeva AY , Bouasker S, Simard MJ, Hammell CM, Ambros V. 2014. Mutations in conserved residues of the *C. elegans* microRNA Argonaute ALG-1 identify separable functions in ALG-1 miRISC loading and target repression. PLoS Genet. 10(4):e1004286. doi:10.1371/journal.pgen.1004286. 24763381 PMC3998888

[jkae007-B65] Zinovyeva AY , Veksler-Lublinsky I, Vashisht AA, Wohlschlegel JA, Ambros VR. 2015. *Caenorhabditis elegans* ALG-1 antimorphic mutations uncover functions for Argonaute in microRNA guide strand selection and passenger strand disposal. Proc Natl Acad Sci U S A. 112(38):E5271–E5280. doi:10.1073/pnas.1506576112. 26351692 PMC4586838

