# Supplementary material for: An RNAi screen for conserved kinases that enhance microRNA activity after dauer in Caenorhabditis elegans
Source: G3 (Bethesda). 2024 Jan 16;14(3):jkae007. doi: 10.1093/g3journal/jkae007 (PMC10917497; doi:10.1093/g3journal/jkae007)
Supplement: jkae007_Supplementary_Data [file jkae007_supplementary_data.zip › Figure_S1_G3-2024-404825.pdf]

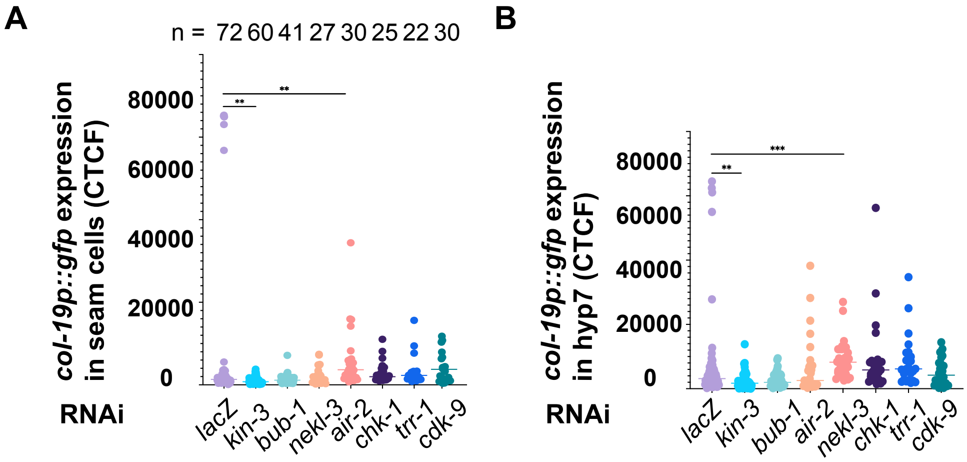

Figure S1. **RNAi of six kinase-encoding genes identified in the primary screen did not reduce *col-19p::gfp* after dauer.** The corrected total cell fluorescence (CTCF) value of *col-19p::gfp* expression in seam cells **(A)** or hyp7 nuclei **(B)** in post-dauer adult animals, as quantified using ImageJ. The CTCF value was calculated by subtracting the mean fluorescence background from the integrated density. Only the *kin-3* control showed reduced *col-19p::gfp* expression in either seam cells or hyp7, compared to *lacZ.* RNAi of *air-2* and *nekl-3* produced slightly increased *col-19p::gfp* expression in seam cells or hyp7, respectively. ** p-value < 0.004, *** p < 0.0008, Kruskal-Wallis and Dunn's multiple comparisons test. Note that when the outliers from the *lacZ* control were removed, *kin-3(RNAi)* treated post-dauer adults still showed significantly reduced *col-19p::gfp* compared to those treated with *lacZ* RNAi. The number of adults scored (n) for each RNAi condition is listed in panel (A). The same animals were scored in panels (A) and (B).
