# Supplementary material for: An RNAi screen for conserved kinases that enhance microRNA activity after dauer in Caenorhabditis elegans
Source: G3 (Bethesda). 2024 Jan 16;14(3):jkae007. doi: 10.1093/g3journal/jkae007 (PMC10917497; doi:10.1093/g3journal/jkae007)
Supplement: jkae007_Supplementary_Data [file jkae007_supplementary_data.zip › Figure S2.pdf]

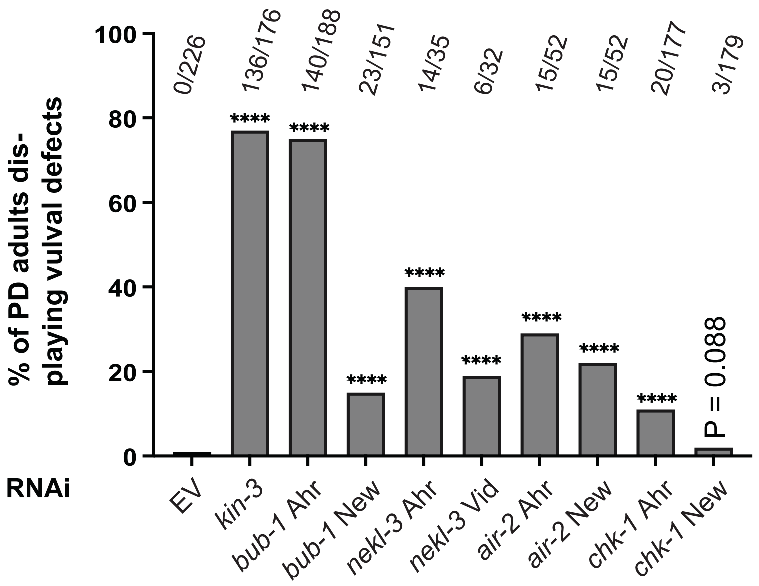

Figure S2. **Two independent RNAi clones of kinases identified in the screen produce vulval defects**. For each gene identified as a hit in the primary and secondary screens, a second RNAi clone was tested for its ability to produce vulval defects (Rup and/or Pvl) in young post-dauer *alg-1(0)* adults. Where available, the second RNAi clone was taken from existing RNAi libraries, "Ahr" = Ahringer library, "Vid" = Vidal library (Kamath and Ahringer 2003; Rual *et al.* 2004). Where unavailable, new RNAi clones were created, "New" (see Methods). *p*-value <0.0001 (****), Fisher exact test compared to EV (empty vector) control.

References:

Kamath RS, Ahringer J. 2003. Genome-wide RNAi screening in *Caenorhabditis elegans*. Methods 30(4):313–321. doi:10.1016/ S1046-2023(03)00050-1.

Rual JF, Ceron J, Koreth J, Hao T, Nicot AS, Hirozane-Kishikawa T, Vandenhaute J, Orkin SH, Hill DE, van den Heuvel S, *et al.* 2004. Toward improving *Caenorhabditis elegans* phenome mapping with an ORFeome-based RNAi library. Genome Res. 14(10b): 2162–2168. doi:10.1101/gr.2505604.
